# Intrauterine growth restriction, prematurity, and low birth weight: risk phenotypes of neonatal death, Rio de Janeiro State, Brazil

**DOI:** 10.1590/0102-311XEN231022

**Published:** 2023-06-26

**Authors:** Pauline Lorena Kale, Sandra Costa Fonseca

**Affiliations:** 1 Instituto de Estudos de Saúde Coletiva, Universidade Federal do Rio de Janeiro, Rio de Janeiro, Brasil.; 2 Instituto de Saúde Coletiva, Universidade Federal Fluminense, Niterói, Brasil.

**Keywords:** Premature Infant, Low Birth Weight, Gestational Age, Early Neonatal Mortality, Survival Analysis, Prematuridade, Baixo Peso ao Nascer, Idade Gestacional, Mortalidade Neonatal Precoce, Análise de Sobrevida, Prematuridad, Recién-Nacido de Bajo Peso, Edade Gestacional, Mortalidad Neonatal Precoz, Análisis de Supervivencia

## Abstract

Intrauterine growth restriction and prematurity determine low birth weight. The combination of the three conditions results in different neonatal phenotypes that interfere with child survival. Neonatal prevalence, survival and mortality were estimated according to neonatal phenotypes in the cohort of live births in 2021 in the state of Rio de Janeiro, Brazil. In this study, live births of multiple pregnancies, with congenital anomalies and inconsistencies in the information of weight and gestational age were excluded. The Intergrowth curve was used to classify weight adequacy. Mortality (< 24 hours, 1-6 and 7-27 days) and survival (Kaplan-Meier) were estimated. In total, 6.8%, 5.5%, and 9.5% of the 174,399 live births were low birth weight, small for gestational age (SGA), and premature, respectively. Considering low birth weight live births, 39.7% were SGA and 70% were premature. The neonatal phenotypes were heterogeneous according to maternal, delivery, pregnancy, and newborn characteristics. The mortality rate per 1,000 live births was high for low birth weight premature newborns, both SGA (78.1) and AGA (adequate for gestational age: 61.1), at all specific ages. Reductions in the survival rate were observed when comparing non-low birth weight and AGA term live births. The estimated prevalence values were lower than those of other studies, partly due to the exclusion criteria adopted. The neonatal phenotypes identified children who were more vulnerable and at higher risk of death. Prematurity contributed more to mortality than SGA, and its prevention is necessary to reduce neonatal mortality in the state of Rio de Janeiro.

## Resumo

A restrição do crescimento intrauterino e a prematuridade determinam o baixo peso ao nascer, e a combinação das três condições resulta em diferentes fenótipos neonatais que interferem na sobrevivência infantil. Foram estimadas a prevalência, a sobrevivência e a mortalidade neonatal, segundo os fenótipos neonatais, na coorte de nascidos vivos de 2021 no Rio de Janeiro, Brasil. Foram excluídos nascidos vivos de gravidez múltipla, com anomalia congênita, e com inconsistências nas informações de peso e idade gestacional. Foi utilizada a curva Intergrowth para classificar adequação do peso, e estimadas a mortalidade (< 24 horas, 1-6 e 7-27 dias) e sobrevida (Kaplan-Meier). Dos 174.399 nascidos vivos, 6,8%, 5,5% e 9,5% eram, respectivamente, baixo peso ao nascer, pequeno para idade gestacional (PIG) e prematuros. Entre nascidos vivos com baixo peso ao nascer, 39,7% eram PIG e 70%, prematuros. Os fenótipos neonatais foram heterogêneos segundo características maternas, do parto, da gestação e do recém-nascido. A taxa de mortalidade por 1.000 nascidos vivos foi elevada para neonatos de baixo peso ao nascer prematuros, tanto PIG (78,1) quanto AIG (adequado para idade gestacional: 61,1), em todas as idades específicas. Houve reduções significantes da sobrevida quando comparados aos nascidos vivos não baixo peso ao nascer, AIG termo. As prevalências estimadas mostraram menores valores que as de outros estudos, em parte pelos critérios de exclusão adotados. Os fenótipos neonatais identificaram crianças mais vulneráveis e com maior risco de morte. A prematuridade contribuiu mais para a mortalidade que a condição de PIG; sua prevenção é necessária para reduzir a mortalidade neonatal no Estado do Rio de Janeiro.

## Resumen

La restricción del crecimiento intrauterino y la prematuridad determinan el bajo peso al nacer, y la combinación de las tres condiciones da como resultado diferentes fenotipos neonatales que interfieren en la supervivencia infantil. Se estimó la prevalencia, supervivencia y mortalidad neonatal según los fenotipos neonatales, en la cohorte de nacidos vivos en 2021 en el Estado de Río de Janeiro, Brasil. Se excluyeron nacidos vivos de embarazo múltiple, con anomalía congénita y con inconsistencias en la información sobre el peso y edad gestacional. Se utilizó la curva Intergrowth para clasificar la adecuación de peso, y se estimó la mortalidad (< 24 horas, 1-6 y 7-27 días) y supervivencia (Kaplan-Meier). De los 174.399 nacidos vivos, 6,8%, 5,5% y 9,5% fueron, respectivamente, bajo peso al nacer, pequeños para la edad gestacional (PIG) y prematuros. Entre los bacidos vivos com bajo peso al nacer, el 39,7% eran PIG y el 70% prematuros. Los fenotipos neonatales fueron heterogéneos según las características maternas, del parto, del embarazo y del recién nacido. La tasa de mortalidad por 1.000 nacidos vivos fue alta para los neonatos bajo peso al nacer prematuros, tanto PIG (78,1) como AIG (apropiado para la edad gestacional: 61,1), en todas las edades específicas. Hubo reducciones significativas en la supervivencia en comparación con el término AIG bajo peso al nacer nos nacidos vivos. Las prevalencias estimadas mostraron valores inferiores a los de otros estudios, en parte debido a los criterios de exclusión adoptados. Los fenotipos neonatales identificó a los niños más vulnerables y con mayor riesgo de muerte. La prematuridad contribuyó más a la mortalidad que la condición PIG, y su prevención es necesaria para reducir la mortalidad neonatal en el Estado de Río de Janeiro.

## Introduction

Four relevant conditions act as precedents of perinatal and neonatal death: fetal growth restriction, prematurity, congenital anomalies, and asphyxia at the fifth minute - Apgar < 7 ^1^. International studies ^2^ ^,^ ^3^ name them the “Big 4” or “Big 3” (when excluding Apgar). Fetal growth restriction and prematurity deserve more attention since, alone or in combination, they determine low birth weight ^1^ ^,^ ^4^ ^,^ ^5^ ^,^ ^6^ ^,^ ^7^ ^,^ ^8^ ^,^ ^9^.

Ashorn et al. ^4^ proposed the analysis of mortality risks of more vulnerable newborns, considering birth weight, prematurity and weight adequacy for gestational age in different phenotypes, according to their combination.

Birth weight remains one of the most important markers of maternal and child health, and 2,500g remains the cutoff point to define low birth weight ^10^. The World Health Organization (WHO) ^11^ proposed a 30% reduction births with low weight by 2025, however, the 2015 evaluation showed slow progress towards this goal ^10^. About 20 million births with low weight were estimated, resulting in a 14.6% prevalence, with regional differences ^10^. It is thus a complex event, and efforts to identify its proximal determinants - prematurity and fetal growth restriction - are recommended ^11^.

In 2014, the estimated prevalence of prematurity - births under 37 weeks of gestational age -worldwide was 10.6%, ranging from 8.4% in Europe to 13.4% in North Africa, and 9.8% in Latin America ^12^. In 2019, a slight drop to 10.2% was observed worldwide ^5^. In Brazil, the estimate from 2011 to 2018 was 9.4% ^9^. On the other hand, the emergence of COVID-19 led to an increase in preterm births and severe morbidity and mortality of the mother and child binomial due to the association with maternal infection, in a cohort of pregnant women in early 2020 ^13^, however, such results are not uniform ^14^.

Regarding fetal growth restriction, the prevalence of infants small for gestational age (SGA) is often used as proxy, considering the 10th percentile of growth curves as the cutoff. The estimate for low- and middle-income countries, in 2012, based on the Intergrowth curve, was 19.3%, with 34.2% as the highest value, found in South Asia ^8^. Latin America had a prevalence of 8.6% and Brazil of 9% ^8^. The *Birth in Brazil* study, using data from a similar period and population percentiles developed with the data itself, reported 11.1% prevalence ^15^. However, another national evaluation, from 2011 to 2018, using percentiles of the Intergrowth curve that restricts the population to the range of 24 to 42 weeks, reported a 9.2% prevalence ^9^.

Social and health care inequities such as unfavorable socioeconomic conditions, absence of a partner, extremes of age, non-white skin/color, low maternal schooling, smoking, newborn with previous low weight, hypertensive syndrome during pregnancy, chronic morbidities (lupus and kidney disease), low gestational weight gain, and inadequate prenatal care were associated with fetal growth restriction in the country ^15^ ^,^ ^16^. In *Birth in Brazil*, the highest attributable fractions were nulliparity, hypertensive syndrome during pregnancy, low gestational weight gain, and smoking ^15^. Many of the factors for SGA birth are also related to prematurity, especially extremes of maternal age, low schooling, indigenous ethnicity, black skin color, and absence of a partner ^9^. Furthermore, cesarean section is implicated in the increase of preterm birth prevalence, even after adjusting for maternal characteristics such as age, schooling, marital status and parity ^17^.

This study estimated the prevalence of low birth weight, fetal growth restriction, prematurity, as well as survival rates and specific neonatal mortality by age according to these phenotypes in the 2021 live birth cohort in the state of Rio de Janeiro, Brazil.

## Methodology

This is a retrospective cohort study of live births in 2021 in the state of Rio de Janeiro followed from birth to 27 full days of life. The outcome was specific neonatal death (< 24 hours, 1-6 days, 7-27 days) ^18^ from 2021 to 2022.

Data were obtained from the Brazilian Information Systems on Live Births (SINASC - 2021: 189,945 live births) and Brazilian Mortality Information System (SIM - neonatal deaths of newborns in 2021 and occurred from January 1st, 2021, to January 27th, 2022: 1,627) of the Rio de Janeiro State Health Department. The databases, provided in June 2022, in physical digital format (CD-ROM), without nominal and residential identification, were deterministically related using number of the Declaration of Live Birth as key variable. The losses were about 12% and not selective regarding age at death and maternal and neonatal characteristics ^19^.

Eligible newborns had birth weight ≥ 500g, gestational age ≥ 22 weeks, and were children of single pregnancy. Records with values of inconsistent weight for gestational age, that is, values outside the consistent ranges of the lowest 3rd percentile value and the highest 97th percentile value ^19^, and with reports of congenital anomalies (10th revision of the International Classification of Diseases - ICD-10 - code described in field 34 of the Declaration of Live Births), were excluded, due to their relationship with birth weight, growth restriction, and prematurity ^9^.

To classify the newborn according to fetal growth, the adequacy of birth weight for gestational age was used, considered a proxy for this estimate. Although the use of adequacy of weight is criticized for not presenting a perfect correspondence with fetal growth ^20^ ^,^ ^21^, it is a relatively accessible and pragmatic measure. The newborn was defined as having adequate weight for gestational age (AGA: 10th-90th percentiles), small for gestational age (SGA: < 10th percentile), and large for gestational age (LGA: > 90th percentile), using gender-specific curves from the INTERGROWTH-21st ^22^ ^,^ ^23^. This methodology does not consider gestational age of 22 and 23 weeks or greater than 42 weeks. Thus, records with these values were excluded from the analysis.

For the following analyses, in which growth restriction and prematurity were the variables of interest, LGA newborns were excluded. The resulting population was classified as: (1) birth weight - not low birth weight (≥ 2,500g) and low birth weight (< 2,500g); (2) gestational age - term (≥ 37 weeks) and prematurity (< 37 weeks); (3) adequacy of weight for gestational age - AGA and SGA. The resulting combinations were eight: (a) not low birth weight (term AGA, preterm AGA, term SGA, and preterm SGA), (b) low birth weight (term AGA, preterm AGA, term SGA, and preterm SGA). The reference category was not low birth weight term SGA, due to the lower risk of death.

After classification, live births were described according to maternal characteristics. Sociodemographic variables included age group (10-19, 20-34 and ≥ 35 years old); skin color (white, black and brown), and schooling (0-3; 4-7 and ≥ 8 years of study). Parity was evaluated according to the number of previous deliveries (zero: primiparous and ≥ 1: multiparous). The adequacy of access to prenatal care ^19^ was analyzed in a dichotomous way (no prenatal care and beginning of prenatal care ≥ 4th month or beginning of prenatal care ≤ 3rd month); as well as the mode of delivery (vaginal and cesarean section). Characteristics of the newborn were also evaluated: cephalic presentation at the time of delivery (yes and no), gender (female and male), and fifth minute Apgar score (< 7 and ≥ 7).

### Statistical analyses

Absolute and percentage distributions of the variables, adequacy of weight and gestational age, prematurity, and low weight were described for neonatal survivors and by age at death. Mean and the respective 95% confidence intervals (95%CI) were calculated for the variables weight and gestational age according to phenotype. Pearson’s chi-square test, Fisher’s exact test and ANOVA (statistical significance of 5%) were used. The rate of specific neonatal mortality by age *i* (NMR - quotient between number of neonatal deaths at age *i* by the number of live births in 2021) per 1,000 live births, the relative risks and the 95%CI according to the combinations of low weight, prematurity, and adequacy of weight for gestational age were estimated.

The Kaplan-Meier method was used to analyze the survival curves ^24^ of newborns according to low weight, adequacy of weight and gestational age, and prematurity, as well as the resulting combinations. The survival time (day) was calculated by the difference between the date of death or censoring due to the end of the neonatal follow-up period (27 days) and the date of birth. Neonatal deaths in less than 24 hours were considered as contributing (0.5 day) to the survival estimates. To test the difference between the survival curves, the log-rank statistical test (5% statistical significance) was used. The computer program used was Stata SE, version 12 (https://www.stata.com).

This study was approved by the Ethics Research Committee of the Fluminense Federal University (n. 29721320.0.0000.5243, opinion 4.091.556, from June 16^th^, 2020).

## Results

**Figure 1:**
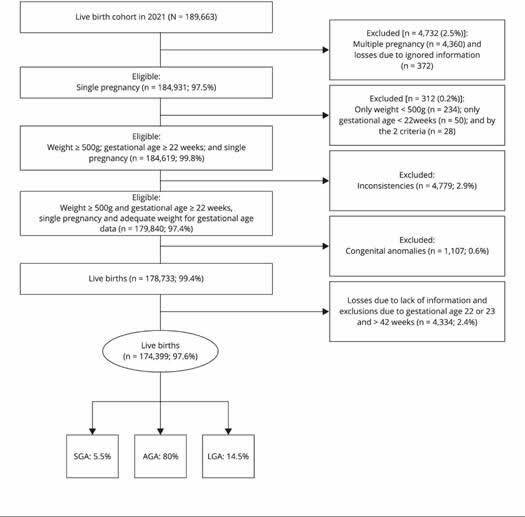
Diagram of the live birth cohort of 2021, state of Rio de Janeiro, Brazil.

AGA: adequate for gestational age; LGA: large for gestational age; SGA: small for gestational age. Source: Brazilian Information Systems on Live Births from the Rio de Janeiro State Health Department (databases provided in June 2022 in physical digital format - CD-ROM).

The 2021 cohort consisted of 189,663 live births, of which 178,733 were eligible. The prevalence rates of SGA and LGA newborns were, respectively, 5.5 and 14.5% (Figure 1).

The prevalence rates of preterm births were: 9.5% for the total, 13.2% for SGA live births, 8.8% for AGA live births, and 13.7% for LGA live births. Regarding low weight, the prevalence rates were: 6.8% for the total, 39.7% for SGA, 5.6% AGA, and 1.1% LGA. Among live births with low birth weight, 70% were premature.

Live births classified in the categories of birth weight, prematurity, SGA, and AGA were heterogeneous according to all maternal sociodemographic characteristics, pregnancy, delivery, and newborn (p < 0.0001) (Table 1). Among the newborns weighing ≥ 2,500g (not low birth weight) and no SGA live births was classified as preterm. Thus, this category was excluded from the analyses.

The highest proportions of mothers ≥ 35 years old were among preterm live births, regardless of the adequacy of weight for gestational age. On the other hand, the highest proportion of adolescents were among not low birth weight and SGA full term live births. The lowest schooling level was observed among mothers of SGA and term live births, both low birth weight and not low birth weight. Black skin color presented a higher percentage between SGA with low birth weight and preterm live births. Inadequate prenatal care predominated among low birth weight live births, especially SGA and preterm. Cesarean section and non-cephalic presentation were mostly observed in preterm live births, especially with low birth weight and SGA. Asphyxia occurred mostly in preterm live births, with low weight, regardless of adequacy of weight and age. Boys predominated among preterm and girls among low birth weight live births (Table 1).

**Table 1:** Maternal and live birth characteristics classified according to the conditions: adequacy of weight and gestational age, prematurity and low birth weight. Live birth cohort of 2021, state of Rio de Janeiro, Brazil.

Characteristics *	Not low birth weight						Low birth weight							
	AGA				SGA			AGA				SGA			
	Term		Preterm		Term		Term		Preterm		Term		Preterm	
	n	%	n	%	n	%	n	%	n	%	n	%	n	%
Age group (years)														
< 20	16,187	12.8	705	13.1	1,032	17.9	141	15.3	1,007	14.6	400	15.8	172	13.6
20-34	89,205	70.6	3,501	64.8	3,987	69.0	608	65.9	4,383	63.6	1,659	65.6	796	62.8
≥ 35	20,977	16.6	1,197	22.2	759	13.1	174	18.9	1,497	21.7	471	18.6	299	23.6
Schooling level (years fos study)														
0-3	1,302	1.1	72	1.4	85	1.5	11	1.2	89	1.3	53	2.1	19	1.5
4-7	16,536	13.4	745	14.1	989	17.6	127	14.0	992	14.8	469	18.9	182	14.7
≥ 8	105,755	85.6	4,454	84.5	4,554	80.9	768	84.8	5,626	83.9	1,957	78.9	1,038	83.8
Skin color														
White	40,311	32.9	1,824	34.9	1,469	26.2	307	34.0	2,153	32.2	719	29.3	333	27.2
Black	17,944	14.6	775	14.8	1,004	17.9	148	16.4	1,042	15.6	440	17.9	241	19.7
Brown	64,462	52.5	2,626	50.3	3,135	55.9	447	49.6	3,497	52.3	1,299	52.9	651	53.1
Previous deliveries														
0	54,080	43.5	2,297	43.1	2,869	50.5	460	51.0	3,261	48.1	1,232	49.7	654	52.4
≥ 1	70,281	56.5	3,034	56.9	2,809	49.5	442	49.0	3,523	51.9	1,249	50.3	594	47.6
Prenatal														
Did not or started ≥ 4th month	26,841	21.4	1,206	22.5	1,458	25.4	213	23.0	1,770	25.6	637	25.1	339	26.3
Beginning ≤ 3rd month	98,656	78.6	4,167	77.55	4,283	74.6	715	77.1	5,144	74.4	1,897	74.9	950	73.7
Mode of delivery														
Vaginal	56,495	44.7	2,093	38.8	3,017	52.2	373	40.4	2,538	36.9	1,152	45.6	349	27.6
Cesarean section	69,800	55.3	3,308	61.3	2,758	47.8	550	59.6	4,347	63.1	1,377	54.5	916	72.4
Cephalic presentation														
Yes	121,480	97.6	5,113	95.9	5,534	97.3	877	96.2	6,193	91.7	2,399	96.3	1,107	89.4
No	3,038	2.4	218	4.1	153	2.7	35	3.8	563	8.3	92	3.7	132	10.7
Sex														
Female	61,932	49.0	2,199	40.7	2,601	45.0	587	63.6	3,447	50.1	1,497	59.2	648	51.1
Male	64,437	51.0	3,204	59.3	3,177	55.0	336	36.4	3,440	50.0	1,033	40.8	619	48.9
Apgar (fifth minute)														
< 7	702	0.6	47	0.9	52	0.9	10	1.1	373	5.5	13	0.5	65	5.3
≥ 7	124,558	99.4	5,309	99.1	5,633	99.1	899	98.9	6,398	94.5	2,454	99.5	1,171	94.7

AGA: adequate for gestational age; SGA: small for gestational age.

Source: Brazilian Information Systems on Live Births from the Rio de Janeiro State Health Department (databases provided in June 2022 in physical digital format - CD-ROM).

Note: low birth weight (< 2,500g); not low birth weight (≥ 2,500g); preterm (< 37 weeks), term (≥ 37 weeks).

* The phenotypic groups were heterogeneous according to all characteristics analyzed (Pearson’s chi-square test p < 0.0001).

Mean weight and gestational age showed significant differences between the seven phenotypes analyzed. Particularly, for the two phenotypes of term and low weight live births, the mean gestational age of SGA live births was about one week higher than AGA live births (p < 0.00001) (Table 2). Low birth weight live births, term and AGA were 37 gestational weeks. Among low birth weight live births, term and SGA, gestational age ranged between 37 and 40 weeks, with the highest frequency, at the 39th week (47.4%), when it reaches 78.8% of the entire distribution Supplementary Material https://cadernos.ensp.fiocruz.br/static//arquivo/supl-een231022_3491.pdf).

**Table 2:** Mean gestational age (weeks) and birth weight (grams) by components according to prematurity, low weight, and adequacy of weight and gestational age. Live birth cohort of 2021, state of Rio de Janeiro, Brazil.

Live births	Gestational age *		Weight *	
	Average	95%CI	Average	95%CI
Not low birth weight				
AGA term	39.0	38.9; 39.0	3,222.4	3,220.8; 3,224.1
AGA preterm	35.7	35.7; 35.7	2,787.2	2,782.0; 2,792.3
SGA term	39.8	39.8; 39.9	2,692.5	2,689.2; 2,695.8
Low birth weight				
AGA term	37.0	-	2,426.5	2,423.6; 2,429.4
AGA preterm	33.2	33.1; 33.3	1,951.5	1,940.2; 1,692.8
SGA term	37.9	37.9; 37.9	2,356.5	2,352.1; 2,360.9
SGA pretermo	33.9	33.8; 34.1	1,618.8	1,594.6; 1,643.0

95%CI: 95% confidence interval; AGA: adequate for gestational age; SGA: small for gestational age.

Source: Brazilian Information Systems on Live Births from the Rio de Janeiro State Health Department (databases provided in June 2022 in physical digital format - CD-ROM).

Note: low birth weight (< 2,500g); not low birth weight (≥ 2,500g); preterm (< 37 weeks), term (≥ 37 weeks).

* Analysis of variance - ANOVA (p < 0.00001).

The analysis of neonatal mortality showed higher rates for preterm newborns with low birth weight, both SGA and AGA, at all specific ages of neonatal death. In the not low birth weight group, prematurity increased mortality rates more than SGA. The highest risk of neonatal death was among SGA preterm newborns with low birth weight (78.1 per 1,000 live births), followed by AGA preterm newborns with low birth weight (61.1 per 1,000 live births). Regarding the specific age at death, SGA preterm newborns with low birth weight had the highest mortality from 1 to 6 days (47.4 per 1,000 live births), and AGA preterm newborns with low birth weight from 7 to 27 days (20.2 per 1,000 live births). All combinations showed high mortality rates when compared to the not low birth weight AGA category. The highest relative risk (RR = 79) was observed for mortality from 1 to 6 days of preterm SGA low birth weight libe births (Table 3).

**Table 3:** Mean gestational age (weeks) and birth weight (grams) by components according to prematurity, low weight, and adequacy of weight and gestational age. Live birth cohort of 2021, state of Rio de Janeiro, Brazil.

Live births	Neonatal mortality rate											
	< 24 hours			1-6 days			7-27 days			Neonatal		
	%	RR	95%CI	%	RR	95%CI	%	RR	95%CI	%	RR	95%CI
Nort low birth weight												
AGA term	0.2	1.0	-	0.6	1.0	-	0.5	1.0	-	1.3	1.0	-
AGA preterm	1.1	5.5	2.1; 12.6	2.4	4.0	2.3; 7.6	2.0	4.0	2.1; 7.5	5.6	4.0	2.9; 6.3
SGA term	0.2	1.0	0.1; 6.0	1.9	3.2	1.8; 6.3	1.2	2.4	1.1; 5.1	3.3	2.5	1.6; 4.1
Low birth weight												
AGA term	1.1	5.5	0.7; 37.3	2.2	3.7	0.9; 15.5	1.1	2.2	0.3; 15.2	4.3	3.3	1.2; 9.0
AGA preterm	13.4	67.0	40.7; 96.0	27.6	46.0	37.0; 63.4	20.2	40.4	29.3; 52.6	61.1	47.0	39.4; 56.3
SGA term	1.2	6.0	1.7; 18.3	0.8	1.3	0.3; 5.7	2.0	4.0	1.55; 9.5	4.0	3.1	1.6; 5.8
SGA preterm	11.0	55.0	27.2; 98.4	47.4	79.0	59.3; 116.5	19.7	39.4	24.3; 60.6	78.1	60.1	47.2; 76.8

95%CI: 95% confidence interval; AGA: adequate for gestational age; SGA: small for gestational age.

Source: Brazilian Information Systems on Live Births and Brazilian Mortality Information System from the Rio de Janeiro State Health Department (databases provided in June 2022 in physical digital format - CD-ROM).

Note: low birth weight (< 2,500g); not low birth weight (≥ 2,500g); preterm (< 37 weeks), term (≥ 37 weeks).

**Figure 2:**
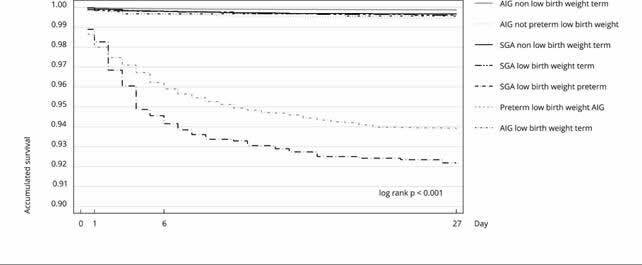
Neonatal cumulative survival curve according to phenotypes based on weight adequacy for gestational age, as well as prematurity and low birth weight. Live birth cohort of 2021, state of Rio de Janeiro, Brazil.

AGA: adequate for gestational age; SGA: small for gestational age. Note: low birth weight (< 2,500g); not low birth weight (≥ 2,500g); preterm (< 37 weeks), term (≥ 37 weeks).

Neonatal survival rate was higher than 92% in the live birth cohort. The survival proportions of low birth weight and not low birth weight newborns were, respectively, 94.74% (95%CI: 94.32; 95.13) and 99.85% (95%CI: 99.82; 99.86); preterm and term, 95.41% (95%CI: 95.05; 95.75) and 99.85% (95%CI: 99.83; 99.87); and SGA and AGA, 98.66% (95%CI: 98.41; 98.87) and 99.55% (95%CI: 99.52; 99.59) The smallest difference in estimated survival was between AGA and SGA live births (0.84%) and about 5% between term and preterm live births, and not low birth weight and low weight. When the three conditions are combined, significant reductions in neonatal survival are observed, especially for preterm newborns with low birth weight, both for AGA and SGA, the latter being the one with the lowest survival, when compared to not low birth weight and AGA term newborns (p < 0.001). Figure 2 shows the survival of the phenotypes. Preterm SGA live births with low birth weight only presented a higher survival rate than the preterm AGA live births with low birth weight in the first two days of life, although the values were close. Then, survival values are distant, always higher for preterm AGA live births with low birth weight (Figure 2).

## Discussion

In the cohort of live births from the state of Rio de Janeiro, in 2021, excluding congenital anomalies, the prevalence of prematurity was 9.8%, 6.8% for low birth weight, and 5.5% for SGA. The risk of neonatal death was more influenced by prematurity than by fetal growth restriction, regardless of the phenotypes analyzed. The highest risk of neonatal death occurred in the combination of the three conditions - low birth weight, SGA and preterm live births.

The prevalence of SGA newborns (without exclusion criteria and using the Intergrowth curve) was equal to the one of this study in the cohort of pregnant women from 2009 to 2012 in the capital of Rio de Janeiro ^25^. However, the proportional distribution of phenotypes differs from other Brazilian studies, which estimated higher values for low birth weight and SGA, and similar values for prematurity ^9^ ^,^ ^15^. Souza et al. ^15^ used data from *Birth in Brazil* - a survey based on hospital records, between 2011 and 2012 - and estimated the adequacy of weight for gestational age by a curve generated with the data, without using exclusion criteria for live births. Paixão et al. ^9^ evaluated SINASC and SIM data from 2011 to 2018, using the Intergrowth curve (≥ 24 and < 43 weeks) and excluding live births with weights considered implausible (< 350g or > 6,500g). Applying the criteria of Paixão et al. ^9^ to the 2021 cohort data of the present study, the prevalence rates of prematurity, low weight, and SGA would increase to 10.4%, 8.4%, and 6.7%, respectively (data not presented in the tables), but would still be slightly different. Thus, factors, probably populational, may interfere in the distribution of phenotypes. In the cohort of Brazilian live births of single pregnancy, without congenital anomaly, and extremely poor (2012/2015) ^16^, the prevalence of SGA (Intergrowth) was 7.8%, the highest. The predominance of prematurity over low weight in the profile of newborns confirms what a study observed in Pelotas (Rio Grande do Sul State) and Argentina in the early 2000s ^26^. The constitution of live births with low birth weight was also similar, totaling 70% of preterm live births.

Among the factors that may affect the comparability of phenotype studies are population and methodological factors. The population distribution of gestational ages, birth weight, and fetal growth restriction varies between countries and regions within a same country ^8^ ^,^ ^10^ ^,^ ^11^ ^,^ ^12^ ^,^ ^26^ ^,^ ^27^. In the study of the Child Health Epidemiology Reference Group SGA - Preterm Birth Working Group, the prevalence of the low birth weight, preterm and SGA phenotype was 0.7% in Latin America, 1.6% in Africa, and 2.3% in Asia ^27^. Regarding the methodological choices, the factors include different diagnostic approaches to gestational age ^28^, different curves to assess the adequacy of weight for gestational age ^22^ ^,^ ^29^ ^,^ ^30^, the type of basis of the study - population or hospital - the inclusion and exclusion criteria of newborns, especially at risk, such as twins and those with congenital malformations, who usually have higher frequencies of low weight, prematurity, and fetal growth restriction ^31^ ^,^ ^32^ ^,^ ^33^. Notably, in Brazil, most deliveries occur in hospitals, which would be less relevant for national studies ^15^.

Although the prevalence rates in the state of Rio de Janeiro were different from those of the study by Paixão et al. ^9^ - both using Intergrowth percentiles, although with different newborn weight restrictions, respectively, below 500g and 350g - the behavior of each phenotype regarding mortality was very similar. In the group of low birth weight live births term, AGA showed higher mortality rate than SGA. This can be explained by the difference in gestational ages: although all were full-term infants, the AGA group was composed only of preterm live births (37 weeks) and were about one week less a olhada than the SGA group Material Suplementar: https://cadernos.ensp.fiocruz.br/static//arquivo/supl-een231022_3491.pdf). Preterm newborns have a higher risk of morbidity and mortality than full-term newborns with a gestational age greater than 39 weeks (not preterm) ^34^.

In African countries, prematurity, alone or combined with fetal growth restriction, presented a higher risk of infant mortality, especially in the neonatal component ^6^ ^,^ ^7^. Wilcox et al. ^21^ tested the validity of low weight, prematurity, and SGA separately to predict neonatal mortality, from the estimation of the area under the ROC curve, concluding that only prematurity presented a good performance to identify at-risk newborns. In the cohort of Danish live births of single pregnancy, weight ≥ 500g, and ≥ 22 weeks of gestation (1981 to 2015), the hypothesis of a mediating effect of the variables weight and gestational age on the association between low maternal education and neonatal mortality was evaluated. Even in the mediating position, preterm birth seemed to have a greater influence than fetal growth restriction ^35^.

The concentration of deaths of newborns with higher risk phenotypes in the early component of neonatal mortality of this study corroborates the national pattern of the retrospective cohort of live births followed for longer, up to five incomplete years (2011 to 2018) ^9^. In accordance with our results, the greatest impact on neonatal survival rates occurred in the presence of low weight, fetal growth restriction, and prematurity, followed by the phenotypic combination of low weight, AGA, and preterm newborns ^9^. In Ethiopia, by only including live births of low birth weight, Debere et al. ^36^ showed that the largest number of deaths was observed in the preterm and SGA phenotype. Moreover, contrary to our findings, survival rate was lower among the full-term SGA live births with low birth weight than among preterm SGA live births with low birth weight. However, in the Ethiopian study, difficulties in estimating gestational age are described, with 12% of losses due to lack of this information. These two studies also used the Intergrowth curve and the Kaplan-Meier method to estimate survival ^9^ ^,^ ^36^.

Furthermore, the conditions of greater sociodemographic vulnerability were present in the three outcomes. Adolescence and low schooling were more related to fetal growth restriction in full-term infants, corroborating studies in other low- and middle-income countries ^37^ ^,^ ^38^. Older maternal age emerged among preterm live births, according to a study in the city of Rio de Janeiro ^19^. Black people and inadequate prenatal care were frequent in the extreme combination - preterm SGA - which was an expected result since both are corroborated factors for these conditions. However, these results were adjusted and the factors cannot be assumed as determinants.

This study presents some limitations due to losses, since information was either lacking or inconsistent regarding weight for gestational age. A possible lack of measurement accuracy, particularly of gestational age ^39^ and the possible underestimation of SGA with the use of the Intergrowth curve, when compared to other growth curves ^40^ ^,^ ^41^, may have led to the imprecision of some phenotypes analyzed. Notably, other studies identify that, despite the restrictions of this curve, the results of accuracy to predict neonatal mortality, compared to those of other curves, are similar ^42^ ^,^ ^43^.

A strong point of the study was highlighting the representativeness of the results considering the reconstitution of the cohort of live births from the data available in the health information systems that have good national coverage and, mostly, in the state of Rio de Janeiro ^39^. The Intergrowth fetal growth curve is universal and provides greater specificity in the classification of newborns according to the adequacy of birth weight by gestational age, resulting in a lower frequency of false SGA live births ^40^.

Therefore, the estimated prevalence rates of the conditions of risk of low weight neonatal death, SGA, and prematurity showed lower values compared to other studies, partly due to methodological nuances. Furthermore, population differences may have contributed to different phenotypic combinations, as reported in other studies ^26^ ^,^ ^27^.

Phenotypes resulting from the combination of these conditions identified more vulnerable children in the present study, highlighting the contribution of prematurity. Fetal growth restriction and prematurity share causes and consequences and are interlinked in the determination of low weight and, consequently, neonatal morbidity and mortality ^27^ ^,^ ^44^ ^,^ ^45^. There are differences in the profile of newborns at risk according to the distribution of these conditions, which may require different interventions ^27^. In the population studied, the lowest survival rate was in the presence of all three conditions, but prematurity, regardless of the presence of fetal growth restriction, showed greater magnitude, reinforcing its validity as a predictor of neonatal death ^21^, and the need for its prevention for a greater reduction in neonatal mortality in the state of Rio de Janeiro.
