# Supplementary material for: Intrauterine growth restriction, prematurity, and low birth weight: risk phenotypes of neonatal death, Rio de Janeiro State, Brazil
Source: Cad Saude Publica. 2023 Jun 26;39(6):e00231022. doi: 10.1590/0102-311XEN231022 (PMC10547104; doi:10.1590/0102-311XEN231022)
Supplement: Supplementary file 1 [file 1678-4464-csp-39-06-PT231022-s.pdf]

Supplementary Table Mean gestational age (weeks) and birth weight (grams) by components according to prematurity, low weight, and adequacy of weight and gestational age. Live birth cohort of 2021, state of Rio de Janeiro, Brazil.

**Gestational age (weeks)**	**AGA term**		**SGA term**	
	**n**	**%**	**n**	**%**
37	923	100.0	794	31.4
38	0	-	1,199	47.4
39	0	-	513	20.3
40	0	-	24	0.9
41	0	-	0	-
42	0	-	0	-
Total	923	100.0	2,530	100.0
Mean (95%CI)	37.0 (-)		37.9 (37.87; 37.94)	

95%CI: 95% confidence interval; AGA: adequate for gestational age; SGA: small for gestational age.
Source: Brazilian Information Systems on Live Births from the Rio de Janeiro State Health Department (databases provided in June 2022 in physical digital format – CD-ROM).
Note: low birth weight (< 2,500g); not low birth weight (≥ 2,500g); preterm (< 37 weeks), term (≥ 37 weeks).
